# Monitoring and Simulating Environmental Asbestos Dispersion from a Textile Factory

**DOI:** 10.3390/ijerph15071398

**Published:** 2018-07-03

**Authors:** Dongmug Kang, Yongsik Hwang, Yeyong Choi, Se-Yeong Kim, Young-Ki Kim

**Affiliations:** 1 Preventive, Occupational and Environmental Medicine, School of Medicine, Pusan National University, Yangsan 50612, Korea; kangdm@pusan.ac.kr; 2 Occupational and Environmental Medicine, Pusan National University Yangsan Hospital, Yangsan 50612, Korea; oemseyeong@gmail.com; 3 Environmental Health Center, School of Medicine, Pusan National University, Yangsan 50612, Korea; hwangys@pusan.ac.kr; 4 Ban Asbestos Network Korea, Seoul 03039, Korea; choiyy@kfem.or.kr; 5 Environmental Health Center for Asbestos, Pusan National University Yangsan Hospital, Yangsan 50612, Korea

**Keywords:** asbestos monitoring, simulation, export, Indonesia, estimation, exposure, AERMOD, weather conditions, epidemiology

## Abstract

Although workplace asbestos concentrations (AC) have been reported several times, the past environmental AC are relatively poorly studied. Due to the harmful effects of the asbestos industry, production has moved from early industrialized countries (Japan), to late industrialized countries (Korea), and finally to industrializing countries (Indonesia). The purpose of this study was to determine current occupational exposure levels and evaluate neighborhood environmental exposure levels in an Indonesian asbestos textile factory through collaboration among three generation of industrialized countries. Asbestos concentrations were measured inside and outside of the factory and compared with simulated data. ACs in the factory were similar to those of 1980s and 1990s levels in the Korean factory that transferred the machines. Environmental ACs were dispersed according to wind direction. There were no significant differences between monitored and simulated data, and correlation coefficients between downwind, upwind, and middle wind directions were high, with some statistical significance. This study can be used to estimate past environmental ACs to understand the causality of asbestos related diseases. Because of the small sample size and specific weather conditions, a large-scale study of various asbestos exposure sources, including asbestos cement factories, shipyards, and mines, and various atmospheric conditions is required.

## 1. Introduction

Although asbestos related diseases (ARDs) are a significant problem, global asbestos consumption remains high. The World Health Organization (WHO) estimated that 125 million people around the world are occupationally exposed to asbestos, which resulted in 107,000 deaths and 1,523,000 disability-adjusted life years [1]. Between 1994 and 2010, 128,015 and 13,885 deaths were caused by malignant mesothelioma (MM) and asbestosis, respectively, corresponding to 2.18 million (MM) and 180,000 (asbestosis) years of human life lost [2]. Using the worldwide 1994 and 2014 data 15,011 MM deaths per year in recent years were estimated (equivalent to 9.9 deaths per million per year) [3]. Estimated deaths of MM, and global asbestos death based on asbestos use of 2015 data were ranged from 32,373 to 38,400, and from 258,078 to 304,841, respectively [4]. Although worldwide asbestos production and consumption have declined from the 1980 peak (4.8 million t), levels have remained high approximately 1.3 million t in 2016 and 2017 [5]. The current high asbestos consumption can lead to environmental asbestos problems as well as occupational diseases.

Exposure assessment is a crucial element of environmental epidemiology. Because ARDs have long latency periods of 10 to 50 years [6], past exposure data is important to evaluate the causal relationship with current ARDs. Previous studies reported environmental air asbestos levels outside asbestos factories during the 1980s and 1990s [7]. Although the simultaneous measurement of asbestos concentration inside and outside asbestos factories is important for estimating environmental emissions from asbestos exposure sources, it is hard to find asbestos concentration data for both the exposure source (inside the factory) and the surrounding environment. After social concerns related to environmental asbestos health problems, the Korean government promoted the asbestos injury relief act, which compensated environmental asbestos victims without requiring knowledge of environmental exposure level [8].

The asbestos industry is notoriously dangerous [9]. Early industrialized countries (first generation) produced abundant toxic material because the asbestos hazard was not well investigated. After strict regulation in early industrialized countries, the production of toxic material began to move to later industrialized countries (second generation), then newly industrializing countries (third generation). The Japanese company N (first generation) was the largest producer of asbestos materials in Asia in the 1960s. A subsidiary of Japan N and its Korean partner J (second generation) jointly implemented the first asbestos textile plant in Korea in 1971, J Asbestos [9], with the aim of exporting their machinery and importing the processed products [10]. This transfer was repeated from Korean J to company T (third generation) in Indonesia in 1990. Although Japan and Korea have several reports of occupational and environmental ARDs [11,12], no environmental exposure data are available for historical asbestos factories. Although the asbestos industry has involved three generations of industrialized countries, no collaborative studies exist across all three generations. Using an operational textile factory in Indonesia, whose machinery was transferred from Korea, we can simultaneously assess occupational and environmental asbestos exposure levels.

The purpose of this study is to determine current occupational exposure levels and evaluate neighborhood environmental exposure levels in an Indonesian asbestos textile factory. This study will provide information on asbestos concentrations inside and outside of an asbestos textile factory and will be beneficial for researchers, workers, and inhabitants of Indonesia.

## 2. Materials and Methods

### 2.1. Sampling Methods

This joint study was conducted from 26 to 28 August 2008 in Cibinong, Indonesia, by the combined efforts of Japan, Korea, and Indonesia, together with academic experts including occupational and environmental physicians, industrial hygienists, government officers of Indonesia (National Occupational Health and Safety Center), and non-governmental organization members. Ambient air monitoring inside the Indonesian T factory, which was a joint venture with Korean J, was conducted using area and personal sampling methods. Low volume personal air samplers (Gilair3, Gillian Co., Seattle, WA, USA) were used inside the factory with an air speed of 2.0 L/min to a total air volume of 100 L for 50 min. Measurements for each work process were personally taken by the authors from one worker in each work process were conducted over 6 h to produce an 8 h weighted average. According to the method of the National Institute for Occupational Safety and Health (NIOSH), a 7400 membrane cellulose ester filter (pore size 0.8 µm, diameter 25 mm) cassette with a 50 mm cowl was used. Ambient air monitoring with area sampling method was conducted in the middle of the factory for 6 h. The borders of outside factory ambient air asbestos concentrations were determined using a high-volume air sampler (Staplex Co., Brooklyn, NY, USA) with a flow rate of 15.0 L/min to a total volume of 450 L for 30 min. Neighborhood environment sampling was conducted with a high-volume air sampler (Staplex Co., Brooklyn, NY, USA) at a flow rate of 10.0 L/min to a total volume of 1200 L for 120 min. Environmental air sampling points were arranged according to their direction and distance from the factory.

### 2.2. Asbestos Fiber Counting

Asbestos concentrations in air samples were analyzed by phase contrast microscopy (PCM, Nikon 80i, Tyoko, Japan) according to the NIOSH 7400 method. The collected filters were divided into four equal parts, made transparent with acetone, and fixed with Triacetin. The ‘A’ fiber count method (A-Rule: fiber length 5 µm or more and a length-to-diameter ratio of 3:1 or more) was used at 400× magnification, fitted with a Walton-Beckett Graticule (1 field of view 0.00785 mm^2^). A sufficient number of fields of view were counted to reach 100 fibers, which was not more than 100. The sample concentration at each point was calibrated with a blank sample. The blind double check method was conducted by two experts proficient in IHPAT (Industrial Hygiene Proficiency Analytical Testing) and BAPAT (Bulk Asbestos Proficiency Analytical Testing).

### 2.3. Meteorological Data, Emission Estimation, and Comparison between Monitored and Estimated Environmental Asbestos Concentrations

Meteorological data, including wind direction and wind speed, were obtained from the meteorological observation center in Bogor Indonesia, which was the closest meteorological center to the factory. Surface and synoptic daily meteorological data were obtained from Jakarta Soekarno-Hatta, Indonesia [13]. The following information were employed as data for AERMOD (American Meteorological Society Environmental Protection Agency Regulatory Model) [14,15,16]: a wind speed (27 August 2008) of 2.3 m/s; an average wind speed (26–28 August 2008) of 1.96 m/s; an ambient asbestos concentration in the factory of 2.4003 f/cc; an indoor factory volume of 1728 m^2^; a window area of 2.4 m × 0.7 m × 10 each (open window area = 1.2 m × 0.7 m × 12 each = 10.08 m^2^); an estimated intake air volume of 10.08 m^2^ × 2.3 m/s = 23.184 m^3^/s; and an estimated asbestos output from the factory of 23.184 m^3^/s × 2400.3 f/L = 5.56 × 10^7^ f/s = 2.0 × 10^11^ f/h. The monitored and AERMOD simulated asbestos concentrations and latitude and longitude of each site were graphed using the Kriging method with SURFER (ver. 8) software (Goldensoftware, Denver, CO, USA).

Because sample sizes were small and had a non-normal distribution, nonparametric statistical tests were conducted. The observed environmental asbestos concentrations were compared with AERMOD simulations using the Wilcoxon signed rank test and Spearman’s correlation coefficients. Although statistical significances were determined by nonparametric statistical analysis, the results of the parametric test are presented also. Observation data were categorized according to the dominant wind direction: downwind (±45° from factory T and with the dominant wind direction), upwind (±45° from factory T and opposite to the dominant wind direction), and middle (between downwind and upwind). A subgroup analysis according to these wind direction categories was also conducted.

## 3. Results

### 3.1. Production Status and Ambient Asbestos Concentration Inside the Factory

The asbestos textile factory, Indonesian T, consumed 612 kg chrysotile per day, and the monthly raw material consumption was 18 t (85% asbestos, 15% polypropylene and polyester). We confirmed that the same machines traded from Korean J were in operation. Workers had not worn masks at first, before the authors stared to monitor asbestos, and afterwards used cotton masks that did not protect them from the dusts. The production processes, number of machines (MC), and number of workers (WC) were as follows: mixing = 1 MC, 2 WC; carding = 4 MC, 3 WC; spinning = 3 MC, 10 WC; twisting = 2 MC, 4 WC; and weaving = 2 MC, 2 WC. Workers were in three rotating shifts with 21 workers in each shift. Asbestos concentrations of each production process with personal monitoring were as follows: 8.6 f/cc in mixing, 7.3 f/cc in carding, 7.5 f/cc in spinning, 3.9 f/cc in twisting, and 3.1 f/cc in weaving. The mean values (standard deviation: SD) of the personal sampling results was 6.1 (2.4) f/cc. The ambient air asbestos concentration of the factory obtained by area sampling was 2.4 f/cc (Table 1).

### 3.2. Comparison of Monitored and AERMOD Simulation Results of Environmental Ambient Asbestos Concentrations

Table 2 compares the results of monitored environmental ambient air asbestos concentrations according to direction and distance from the factory with those of the AERMOD simulation. Because the ambient asbestos concentration in the factory according to our checks, was 2.4003 f/cc, this value was used for the concentration inside the factory. The highest concentration in the monitored external environment was 5 m south of the factory, at 0.1245 f/cc. The mean values (SDs) of monitoring and simulated data (f/cc) were 0.01587 (0.03919) and 0.00979 (0.01913), respectively. Although the AERMOD concentrations were lower than the monitored concentrations, the differences between them were not significant (Table 2). The mean values were higher in the monitoring data in the downwind direction but higher in the simulated data in upwind and middle wind directions; however, there was no statistical significance.

**Figure 1:**
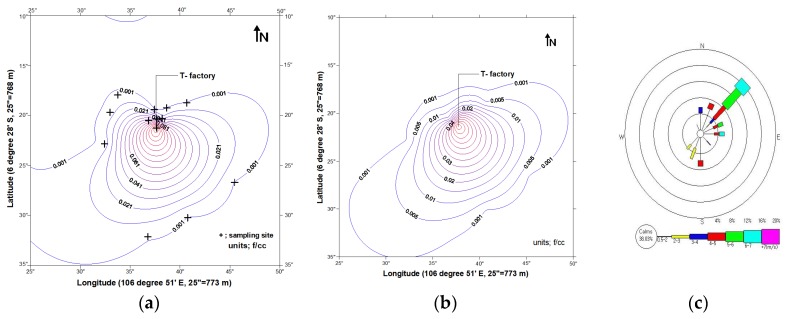
Distribution of asbestos in ambient air according to (**a**) monitoring data, (**b**) AERMOD estimation, and (**c**) a wind rose diagram.

The distribution of asbestos in the ambient air according to the monitoring and AERMOD simulated results are shown in Figure 1. Ambient asbestos was dispersed from the factory according to the wind direction. The distribution of monitoring data was more variable than that of AERMOD simulated data. Contour intervals were closer in the upwind direction and wider in the downwind direction in monitoring data.

**Figure 2:**
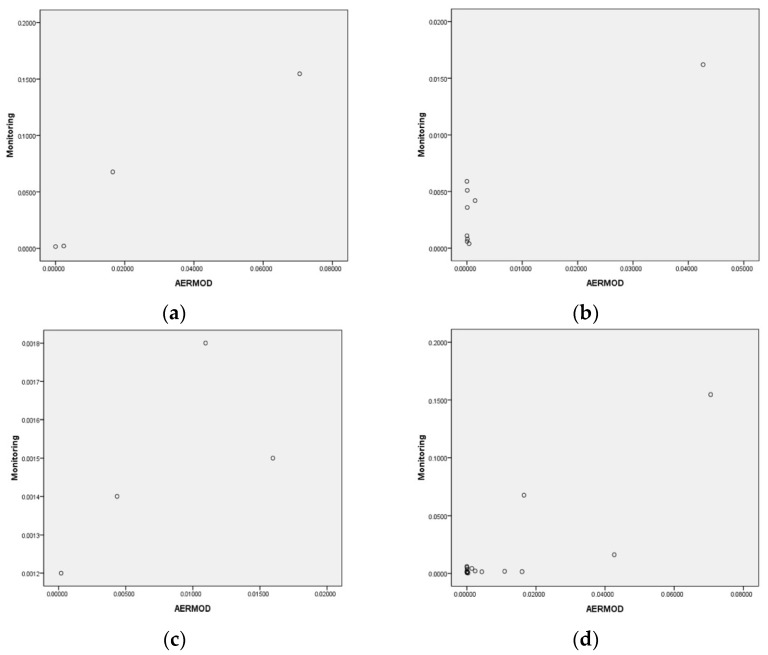
Scatter plots and correlation coefficients between monitoring data and AERMOD simulated data. (**a**) Downwind (Rs = 1.00 *, Rp = 0.96 *); (**b**) middle wind (Rs = 0.84, Rp = 0.91 *); (**c**) upwind (Rs = 0.80, Rp = 0.68); and (**d**) total (Rs = 0.40, Rp = 0.85 *). Rs: Spearman’s correlation coefficient, Rp: Pearson’s correlation coefficient, * *p* < 0.05.

The downwind data sets were highly correlated and statistically significant. The Spearman’s correlation coefficients for middle and upwind directions were high but with no statistical significance. The *p*-values of the Spearman’s correlation coefficients for the down, middle, and up-wind direction, and the total were <0.001, 0.831, 0.200, and 0.112, respectively, while Pearson’s correlation coefficients were high and statistically significant with the exception of the upwind direction (Figure 2). The *p*-values of Pearson’s were 0.025, 0.001, 0.317, and <0.001, respectively. Because we determined statistical significance with nonparametric tests, only the downwind data sets had a statistically significant correlation.

## 4. Discussion

The Indonesian company monitored in this study employs machines and operation methods from Korean J. As Korean J workers in the 1980s and 1990s had improper working conditions and protective devices [10], Indonesian T workers had not worn masks at first, before the authors stared to monitor asbestos, and afterwards used cotton masks that did not protect them from the dust. In this study, asbestos concentrations inside the factory were slightly higher than 1980s Korean J levels and much higher than 1990s Korean J levels (1980s and 1990s means of 4.5 and 0.5 f/cc in mixing, 3.9 and 1.3 f/cc in carding, 5.6 and 2.7 f/cc in spinning, 4.8 and 1.9 f/cc in twisting, and 5.3 and 3.2 f/cc in weaving) [17,18]. The asbestos concentrations sampled personally by the authors from all working processes exceeded the occupational exposure limit of Indonesia, which was 2.0 f/cc of threshold limit values (TLV) [19] until 2011, when it was changed to 0.1 f/cc TLV [20]. The factory used 184 t of chrysotile asbestos annually, which was 0.2% of Indonesia’s total annual consumption (103,445 t) of Indonesia in 2008 [21]. The asbestos consumption level in Indonesia rose until recently [1].

The ambient air asbestos concentration of the Indonesian T factory obtained by ambient air sampling middle of the factory was 2.4 f/cc, which was between that of 1980s (4.4 f/cc) and 1990s (1.7 f/cc) Korean J levels. Thus, the inside factory asbestos concentrations are similar to those of Korean J in the 1980s. The concentrations of environmental ambient asbestos in the downwind direction (0.4–2.1 f/L at 300 m and 0.8–1.1 f/L at 1000 m) are similar to those of a previous study: 2.0 f/L at 300 m and 0.6 f/L at 1000 m [4].

There were no significant differences between monitored and AERMOD simulated data in any wind direction. This suggests that AERMOD can be used to estimate environmental asbestos contamination from asbestos exposure sources when the environmental air conditions and emission levels are known. However, these results might also be explained by the small sample size to have statistical significance. AERMOD tended to simulate lower concentrations with smaller SDs, except in the upwind direction. In general, correlation coefficients between the two data were high, and some were statistically significant. This result also requires careful interpretation because of the non-representative nature of this data. The significant high coefficients according to the Pearson’s correlation test could be influenced by a few very high concentrations. When applying AERMOD and other estimation tools, other meteorological conditions like humidity and other impediments such as buildings between sources and endpoints will likely differ between countries and study period; thus, caution is required when estimating concentrations with simulation tools. In addition, the results of this study might not be an annual representative figure for Indonesia. The study was conducted during the dry season, which has a lower average wind speed (1.96 m/s) than the wet season, which has an average wind speed of 2.71 m/s [22]. Other concerns for applying this study is fiber type. This study deals with chrysotile. Amphiboles including crocidolite have more toxic potency than serpentine asbestos [13,23,24,25]. Also, chrysotile asbestos fiber might have a specific gradient and aerodynamic diameter different to amphibole fibers, which make different dispersion patterns. Hence, the results of this study might not be compatible with other studies with crocidolite and different atmospheric situation such as Japan and U.S. [12,26]. 

The international transfer of asbestos industries among Asian countries has occurred predominantly from Japan and Korea since the 1960s. Asia is one of the highest producers and consumers of asbestos. In 2013, four major countries including Brazil, China, Kazakhstan, and the Russian Federation produced over 99% of the world’s asbestos [1]. In terms of worldwide asbestos consumption, China, India, and the Russian Federation consumed over 60%, and seven countries (Brazil, Indonesia, Kazakhstan, Thailand, Turkmenistan, Uzbekistan, and Vietnam) consumed 30% [1]. Although 8 of the 10 most asbestos consuming countries are in Asia, only a small number of Asian nations i.e., Japan, Korea, and Taiwan [27] have introduced a total ban of asbestos. As the major contributors to ‘pollution export’ and ‘double standards’, Japan and Korea have the responsibility to collaborate with other Asian countries. The fact that the first recognized occupational cancer in Korea was MM in a former worker of Korean J [8] and that the first recognized ARD was asbestosis in an Indonesian J worker (personal communication) is not surprising.

Nonetheless, there are several limitations to this study. First, the sampling equipment at each site could only be checked once so the mean and standard deviation could not be calculated. Although this is an inevitable consequence of a multinational and multi-disciplinary approach, the data in this study may not be valid. However, the agreement between monitoring data, simulated data, and previous measurement data reduces the likelihood of extreme deviations in our data. Second, we analyzed the asbestos fiber with PCM, which might be misclassified, as non-asbestos fibers can be incorporated using the PCM method, which leads to over-calculation. Therefore, to avoid mistaken readings, the sample concentration at each point was calibrated using a blank sample. The blind double check method was conducted by two experts who were proficient in IHPAT and BAPAT. However, over-estimation of PCM is still limitation of this study. However, possible over-estimation of PCM (by about ten percent) is still a limitation of this [28]. Third, this study was conducted on chrysotile only. Because of different toxicity and dispersion characteristics between asbestos fiber types concentration simulation should need caution if fiber type is not given.

We propose that this study is the first to simultaneously determine emissions inside the emission source and at several environmental locations according to wind direction. The results can be used to estimate similar historical environmental exposure. Moreover, this study is among the first conducted by first, second, and third generation countries producing environmentally harmful material. The results and methods of this study can provide information on asbestos concentrations inside and outside of an asbestos textile factory for workers and citizens as well as scholars in Indonesia.

## 5. Conclusions

Similar working conditions and workplace asbestos concentrations were found in an Indonesian asbestos plant and the factory that exported the machines from Korea. Ambient environmental asbestos concentrations were dispersed from the factory according to the wind direction. Considering the lack of historical environmental asbestos exposure data, this research is beneficial for environmental epidemiology studies attempting to reveal the causality of long latency diseases such as ARDs. Considering the small sample size and specific weather conditions of this study, further study of various asbestos exposure sources, including asbestos cement factories, shipyards, and mines, and various atmospheric conditions is required.

## Figures and Tables

**Table 1:** Number of machines and workers, and asbestos concentrations for each production process.

Production Process	Sampling Method (n)	Machine (n)	Worker (n)	Concentration (f/cc)
Mixing	Personal (1)	1	2	8.6
Carding	Personal (1)	4	3	7.3
Spinning	Personal (1)	3	10	7.5
Twisting	Personal (1)	2	4	3.9
Weaving	Personal (1)	2	2	3.1
Ambient air (middle of the factory)	Area (1)			2.4

**Table 2:** Comparison of environmental ambient air asbestos concentration between monitored and AERMOD simulated data according to wind direction from the factory (f/cc) (inside concentration: 2.4003 f/cc).

Wind Direction	Measurement Type	Min	Max	Mean	SD	*p*-Value ^†^	*p*-Value ^‡^
Downwind (*N* = 4)	monitored	0.00160	0.15460	0.05650	0.07239	0.144	0.194
	AERMOD	0.00002	0.07055	0.02238	0.03293		
middle wind (*N* = 9)	monitored	0.00040	0.01620	0.00421	0.00496	0.110	0.820
	AERMOD	0.00001	0.04266	0.00498	0.01414		
Upwind (*N* = 4)	monitored	0.00120	0.00180	0.00148	0.00025	0.144	0.194
	AERMOD	0.00018	0.01597	0.00786	0.00699		
Total (*N* = 17)	monitored	0.00040	0.15460	0.01587	0.03919	0.266	0.331
	AERMOD	0.00001	0.07055	0.00975	0.01913		

^†^ Wilcoxon signed rank test; ^‡^ Paired *t* test.
